# DEPRESSIVE SYMPTOMS MEDIATE THE ASSOCIATION OF FRAILTY PHENOTYPE SYMPTOMS AND COGNITION FOR FEMALES BUT NOT MALES

**DOI:** 10.1093/geroni/igac059.2118

**Published:** 2022-12-20

**Authors:** Nicholas Resciniti, Anwar Merchant, Matthew Lohman

**Affiliations:** University of Southern California, Los Angeles, California, United States; University of South Carolina, Columbia, South Carolina, United States; University of South Carolina, Columbia, South Carolina, United States

## Abstract

We aimed to evaluate whether depressive symptoms mediated the relationship between frailty phenotype and cognitive function by sex. Frailty phenotype symptoms, cognitive function scores, and depressive symptoms were measured in 3,705 community-dwelling US adults from the Health and Retirement Study (HRS) from 20012-2016. Fried’s frailty phenotype criteria (weakness, slowness, physical inactivity, low weight, and exhaustion) were used as a continuous score for frailty symptoms (0-5). Global cognition was measured by the Telephone Interview for Cognitive Status (range: 0-35). The Centers for Epidemiologic Studies-Depression Scale (CES-D) was used as a continuous measure to assess depressive symptoms (0-8). We used mediation analysis to estimate the direct and indirect effects of frailty symptoms on cognitive scores to evaluate the role of depressive symptoms as a potential mediator. Males had a larger total effect (β= -0.43; 95% CI: -0.66, -0.02; p< 0.001) for lower cognitive score for each increase in frailty symptom compared to females (β= -0.28; 95% CI: -0.47, -0.08; p=0.02), suggesting all five frailty symptoms was associated with 2.15 lower cognitive scores for males and 1.50 for females. A significant indirect effect from frailty phenotype to cognition was found through depressive symptoms for females (β= -0.03; 95% CI: -0.06, -0.00; p=0.02) but not males (β= -0.04; 95% CI: -0.08, 0.00; p=0.07). These results highlight the importance of identifying individuals with frailty and depressive symptoms to monitor and provide interventions to preserve cognitive function

